# HAS^4^: A Heuristic Adaptive Sink Sensor Set Selection for Underwater AUV-Aid Data Gathering Algorithm

**DOI:** 10.3390/s18124110

**Published:** 2018-11-23

**Authors:** Xingwang Wang, Debing Wei, Xiaohui Wei, Junhong Cui, Miao Pan

**Affiliations:** 1 Key Laboratory of Symbolic Computation and Knowledge Engineering of Ministry of Education, Jilin University, Changchun 130012, China; 2 College of Computer Science & Technology, Jilin University, Changchun 130012, China; junhong_cui@jlu.edu.cn; 3 Department of Electrical & Computer Engineering, University of Houston, Houston, TX 77204, USA; dwei3@uh.edu (D.W.); mpan2@uh.edu (M.P.)

**Keywords:** underwater data gathering, underwater wireless sensor network, AUV

## Abstract

In this paper, we target solving the data gathering problem in underwater wireless sensor networks. In many underwater applications, it is not quick to retrieve sensed data, which gives us the opportunity to leverage mobile autonomous underwater vehicles (AUV) as data mules to periodically collect it. For each round of data gathering, the AUV visits part of the sensors, and the communication between AUV and sensor nodes is a novel high-speed magnetic-induction communication system. The rest of the sensors acoustically transmit their sensed data to the AUV-visit sensors. This paper deploys the HAS${}^{4}$ (Heuristic Adaptive Sink Sensor Set Selection) algorithm to select the AUV-visited sensors for the purpose of energy saving, AUV cost reduction and network lifetime prolonging. By comparing HAS${}^{4}$ with two benchmark selection methods, experiment results demonstrate that our algorithm can achieve a better performance.

## 1. Introduction

Ocean Big Data (OBD) is an emerging research area that benefits ocean environmental monitoring, offshore exploration, disaster prevention, and military surveillance. Since the oceans, rivers and lakes cover 71% of our planet, and the traditional “deploy, sense, retrieve, and post-process” routine for these sensing activities is difficult and costly for humans in large area applications. Deploying an underwater sensor network is very efficient for monitoring physical factors (e.g., temperature, salty, light) by a multitude of sensing modalities. Since it is impractical to connect every possible underwater sensor by wire, wireless communication is currently the dominant data delivery technique for numerous underwater applications. Current underwater communication fails in three categories, acoustic, electromagnetic and optical [1]. Benefits and drawbacks of these methods are compared in Table 1.

Due to serious attenuation, except for acoustic waves, the effective communication range in sea water is no more than 100 m, which leads to acoustic communication being the dominant data delivery technique for numerous underwater applications. However, acoustic communication is the most difficult obstacle to the realization of these networks, which is caused by limited bandwidth (currently, few hundreds of bits per second), long propagation delay and unchangeable power-battery [2,3,4,5].

**Figure 1:**
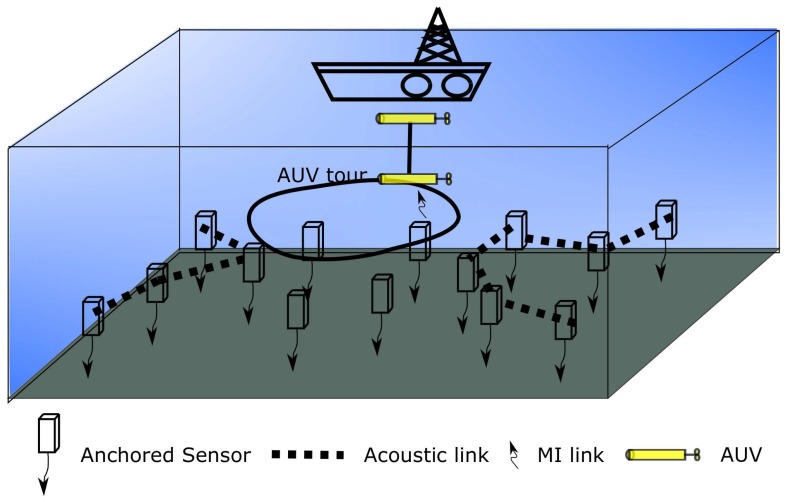
AUV(autonomous underwater vehicles)-aided data gathering.

Fortunately, for underwater environments, magnetic-induction (MI) as an alternative wireless transmission mode besides acoustic communications obtains high bandwidth connectivity (up to 10 Mbps) with energy per-bit orders of magnitude lower than that of acoustic communications [6]. However, for MI, it allows nodes to robustly communicate only when they are a few meters from each other (usually less than 10 m) [7]. Since mobile autonomous underwater vehicles (AUVs) can hover close enough to a sensor node [8,9], using an AUV and multi-modal communications (e.g., acoustic and MI) can enhance the performance of UWSNs and can enable many critical applications. In this scenario, as shown in Figure 1, which leverages the advantages of different wireless underwater transmission modes and the mobility of AUVs, AUVs could be made aware of the sensing task and visit part of the sensors (sink nodes) for data gathering, while the rest of the sensors could acoustically forward their sensing data to sink nodes.

Although AUVs can act as data mules and travel from node to node across a sparsely deployed sensor network to collect data [10], the slow mobility and limited energy that constrain them means that they can only visit a very small number of the sensors. Thus, much sensing data should be acoustically forwarded sensor by sensor. In such a procedure, packets may experience multiple relay nodes before reaching a sink node, which means that an enormous amount of energy is consumed on data forwarding along the path [11]. In addition, with the increasing number of acoustic communications, the probability of package collision and transmission energy consumption could be notably magnified, which will reduce the network performance. Moreover, reducing the total energy consumption and acoustic communications is not enough for prolonging the network lifetime, as some popular sensors, e.g., some neighbours around the sink, may run out of the energy faster than the others, which will cause energy consumption unbalance. Furthermore, due to the channel interference and limitation of acoustic channel capacity, a single sink node may not be able to receive all of the sensing data. Hence, multiple sink nodes are required to collect the whole sensing data. Due to the limitation of AUVs’ energy, sink nodes should be carefully selected.

This paper addresses all of these problems mentioned above to solve the underwater data gathering problem. Targeting the reduction of the total energy consumption on acoustic communication and balancing the energy consumption among the networks, we propose HAS${}^{4}$, a Heuristic Adaptive sink sensor set selection algorithm for underwater AUV-aid data gathering. HAS${}^{4}$ has both centralized and distributed versions. They both achieve energy efficient by decreasing the possible acoustic communication or reducing the maximum acoustic link length. HAS${}^{4}$ prolongs the network lifetime by adaptively selecting short lifetime sensors as sink nodes to eliminate the energy consumption on data transmission.

The contributions of this paper are as follows:

1. We use a mix integer linear problem (MILP) to formulate the underwater AUV-aid data gathering problem. In the formulation, we take the energy consumption on acoustic communication, AUV traveling distance, and energy balance into account to prolong the network lifetime and reduce the AUV traveling cost.
2. We propose HAS${}^{4}$ to solve the MILP (mix integer linear problem). HAS${}^{4}$ is independent from network topologies, and has parameters for trading off between AUV traveling cost and network lifetime. By providing both centralized and distributed versions, HAS${}^{4}$ widens the applicable scenarios.
3. We conduct simulations to verify the efficiency of HAS${}^{4}$. Experiment results show both centralized and distributed HAS${}^{4}$ prolong the network lifetime with low AUV traveling cost.

The remainder of this paper is organized as follows. Section 2 discusses related work. In Section 3, we introduce the problem of AUV-aid underwater data gathering problem. Centralized and distributed algorithms are proposed in Section 4 to solve the problem. Section 5 reports some simulation results. Finally, Section 6 concludes the paper.

## 2. Related Work

Generally, underwater data gathering systems fall into two categories: multi-hop and AUV-aid approaches.

### 2.1. Multi-Hop Approaches

In these approaches, underwater sensing data are forwarded hop-by-hop along routing paths. Thus, routing technique plays the key role in these kinds of approaches. Different from terrestrial wireless sensor networks (WSN), the underwater nodes move with water flows involuntarily in the underwater environment [12,13,14,15,16], and routing protocols designed for WSNs can not be directly applied to UWSNs. Thus, the research focus in this area is to design energy-efficient and reliable routing protocols.

Based on the information required in the protocols, most underwater routing can be classified into location-based and depth-based. Location-based routing protocols, such as vector-based forwarding [17], hop-by-hop vector-based forwarding [18], depth-controlled routing [19], etc., assume that each node knows its location and each packet contains the position of the sender. Depth-based routing protocols, such as depth-based routing [20], directional depth-based routing [21], and depth-based multi-hop routing [22], etc., use depth information to route the packets instead of the full location coordinates.

Due to the energy-extensive consumption of underwater acoustic communication and limited battery power, multi-hop approaches will reduce the lifetime of the network.

### 2.2. AUV-Aid Approaches

In these approaches, AUVs act as data mules to deliver underwater data. Vasilescu et al. illustrated the feasibility of leveraging AUVs for underwater data collection [10]. Tekdas et al. demonstrated the increase of the network lifetime via AUV gathering data from sensor nodes [23].

Research on AUV-aid data gathering has focused primarily on how to reduce the length of AUV’s traveling path [24]. Basagni et al. take the Value of Information (VoI) that value of sensing data decreases over time into account and propose a Greedy and Adaptive AUV Path-planning (GAAP) algorithm [25]. Moreover, the cooperation among AUVs is also studied in 3D underwater environments [26]. However, these works are not using MI communications to improve the reliability and throughput.

In this paper, we leverage the advantages of different wireless underwater transmission modes and the mobility of AUVs to provide a cyber interconnection scheme that enables distributed and efficient data delivery from underwater sensors to the surface stations.

## 3. AUV-Aid Underwater Data Gathering Problem

In this section, we formulate the AUV-aid data gathering problem with the objectives of minimizing the AUV travel distance, minimizing the total energy consumption and maximizing the network lifetime.

### 3.1. Motivation and Overview

**Figure 2:**
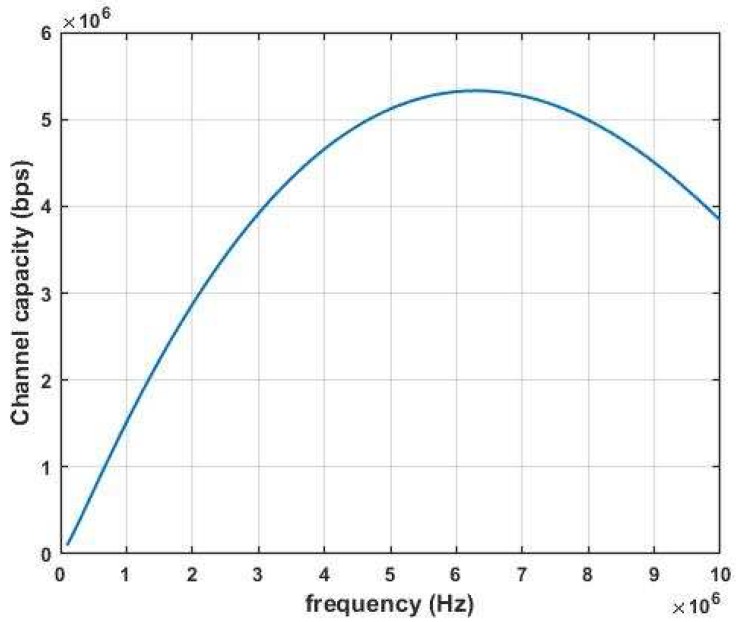
Channel capacity of subsea MI (magnetic-induction) wireless communications with transmission distance = 1 m and coil radius = 0:05 m.

**Figure 3:**
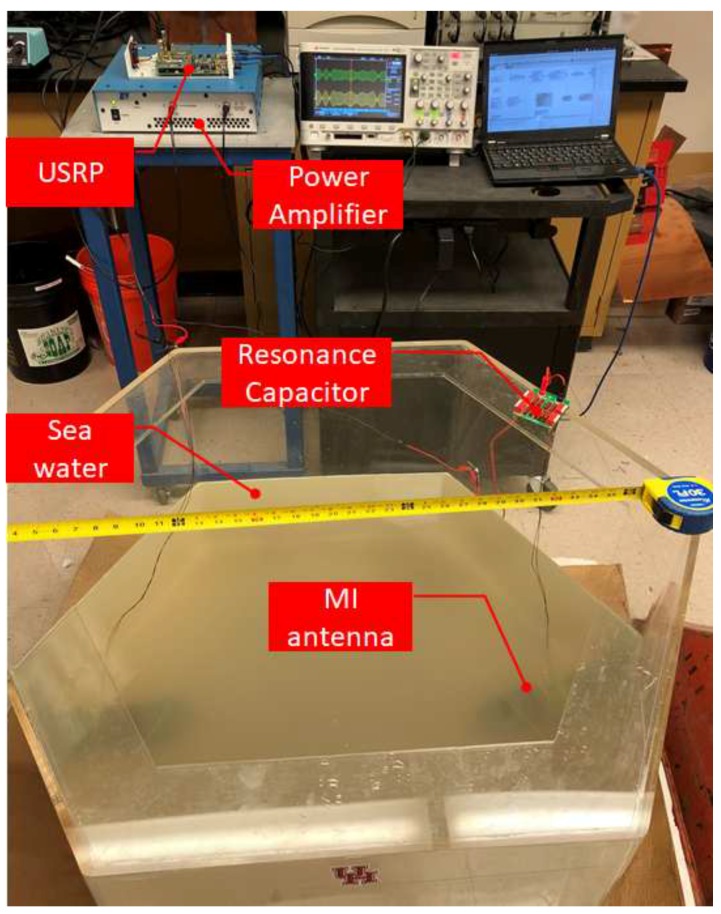
Sub-sea MI communication testbed.

**Figure 4:**
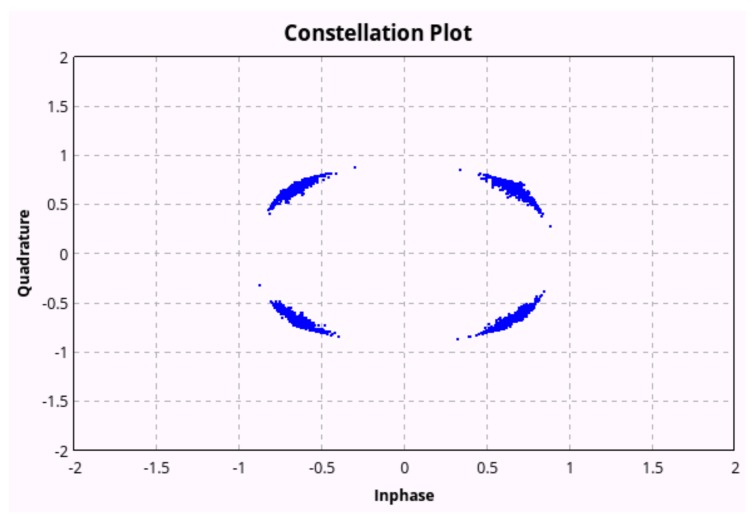
QPSK (quadrature phase shift keying) data transmission results for sub-sea MI (magnetic-induction) wireless communications.

Leveraging the sub-sea MI communication model in [27], as shown in Figure 2, the MI channel capacity can exceed 5 ×$10^{6}$ bps. Moreover, we have built up a sub-sea MI testbed as shown in Figure 3. A data transmission based on Quadrature Phase Shift Keying (QPSK) modulation scheme with symbol rate equal to 100 kHz is demonstrated in Figure 4. Limited by the size of the water tank, we were only able to separate the transmitter and receiver at the distance of 0.7 m. The testing results show that the data transmission is 100% successful. Thus, leveraging the advantages of reliable and high speed MI communication, long distance acoustic communication and the mobility of AUVs, UWSNs can dramatically reduce energy cost on data gathering with high reliability.

Consider an AUV and many sensors form a underwater wireless sensor network. Each sensor is anchored to the target area for ocean monitoring/sensing applications. Both magnetic-induction (MI) and acoustic communication modules are equipped to every sensor for short distance high speed communication and long distance low speed communication, respectively. We assume that the perceived data are periodically gathered and delivered to the designated surface station. During each period, the AUV travels from the surface station, dives into the water and visits a subset of the sensors for data collection, brings the collected data back to the surface station, and prepares for the next round data collection. Since the MI communication range is quite short, the AUV should visit the sensor position exactly for MI communication. These sensors that are not visited by the AUV transmit their sensing data to chosen sensors via acoustical channels.

Suppose there are $S=\{s_{1},s_{2},\cdots ,s_{n}\}$ sensors, and the *i*-th sensor $s_{i}$ is anchored at the coordinate $c_{i}=\left[x^{i},y^{i},z^{i}\right]$. Let $M=\{m_{1},m_{2},\cdots ,m_{k}\}$ denote the acoustic sub-channel set. The transmission range on the *m* acoustic sub channel is $m^{t}$, and the interference range of this channel is $m^{i}$. The neighbor sensor set for each sensor can be obtained according to their coordinates and communication range. For simplicity, in this paper, we assume the AUV’s speed is constant at *V*, and the MI communication rate is constant at $r_{mi}$.

### 3.2. Problem Formulation

As mentioned above, we have three objectives for this problem, and the first objective is to minimize the AUV traveling distance:

(1) $$\min\sum_{i=1}^{n}\sum_{j=1}^{n}d_{ij}x_{ij},$$

where variable $d_{ij}$ is the distance between sensor *i* and sensor *j* and $x_{ij}$ is a binary variable that indicate the AUV travel path. If the AVU travel from sensor *i* to sensor *j*, $x_{ij}=1$. Otherwise, $x_{ij}=0$.

The second objective is to minimize the total energy consumption:

(2) $$\sum_{i=1}^{n}({p_{i}}^{in}\sum_{j\in N_{i}}D_{ji}+{p_{i}}^{out}\sum_{j\in N_{i}}D_{ij}+p_{i}^{out}\left(mi\right)\sum_{j\in N_{i}}D_{ij}),$$

where $D_{ij}$ is the data amount from sensor *i* to sensor *j*. $p_{i}^{out}$ and $p_{i}^{in}$ denote the energy consumption of sensor *i* on transmitting and receiving through acoustic channels, respectively. $N_{i}$ is the neighbour sensor set of sensor *i*.

The final objective is to maximize the network lifetime. We define the network lifetime as the sensing round that the first sensor runs out of energy. Therefore, the objective of maximizing the network lifetime can be seen as maximizing the lifetime that is the minimum lifetime in the network:

(3) $$\max\min\frac{e_{i}}{p_{i}^{0}+p_{i}^{out}\left(mi\right)},\;\forall i\in S,$$

where $p_{i}^{0}$ is the energy consumption on this round predefined sensing task, $e_{i}$ is the residual energy of sensor *i* and $p_{i}^{out}\left(mi\right)$ is the energy consumption on transmitting its own data through MI.

To achieve the above objectives, we have the following constraints:

(4) $$\begin{array}{cc} d_{ij}={∥c_{i}-c_{j}∥}_{2}\; & 1\leq i,j\leq n, \end{array}$$

(5) $$\begin{array}{cc} \sum_{k\in N_{i}}\chi_{ki}^{m}\left(t\right)+\sum_{j\in N_{i}}\chi_{ij}^{m}\left(t\right)\leq 1\; & \forall t,i\in S,m\in M, \end{array}$$

(6) $$\begin{array}{cc} \chi_{ij}^{m}\left(t\right)+\sum_{l\in N_{k}}\chi_{kl}^{m}\left(t\right)\leq 1\; & \forall i\in S,j\in N_{i},k\in I_{m}^{j},k\neq i, \end{array}$$

(7) $$\begin{array}{cc} \chi_{ki}^{m}\left(t\right)\in \left\{0,1\right\}\; & 1\leq i,j\leq n,m\in M, \end{array}$$

(8) $$\begin{array}{cc} \sum_{k\in N_{i}}\chi_{ki}^{m}\left(t\right)=0\; & \forall t,i\in S_{U},m\in M, \end{array}$$

(9) $$\begin{array}{cc} \sum_{i\in N_{k}}D_{ik}=0\; & \forall k\in S_{U}, \end{array}$$

(10) $$\begin{array}{cc} \sum_{i\in N_{k}}\chi_{ki}^{m}\left(t\right)=0\; & \forall t,k\in S_{A},m\in M, \end{array}$$

(11) $$\begin{array}{cc} \sum_{k\in N_{i}}D_{ik}=0\; & \forall i\in S_{A}, \end{array}$$

(12) $$\begin{array}{cc} \sum_{k\in N_{i}}D_{ik}=v_{i}\Delta t\; & \forall i\in S_{U}, \end{array}$$

(13) $$\begin{array}{cc} \sum_{k\in N_{i}}D_{ik}=\sum_{j\in N_{i}}D_{ji}+v_{i}\Delta t\; & \forall i\in S, \end{array}$$

(14) $$\begin{array}{cc} D_{ij}\leq \sum_{t}\sum_{m\in M}c_{ij}^{m}\chi_{ij}^{m}\left(t\right)\; & \forall i,j\in S, \end{array}$$

(15) $$\begin{array}{cc} \begin{array}{c} e_{i}-{p_{i}}^{out}\sum_{j\in N_{i}}D_{ij}-{p_{i}}^{in}\sum_{j\in N_{i}}D_{ji}-\\ p_{i}^{out}\left(MI\right)\sum_{j\in N_{i}}D_{ij}-p_{i}^{0}\Delta t\geq 0 \end{array}\; & \forall i\in S, \end{array}$$

(16) $$\begin{array}{cc} y_{i}d_{i}+\sum_{j\in N_{i}}D_{ij}-v_{i}\Delta t-\sum_{j\in N_{i}}D_{ji}\geq 0\; & \forall i\in S, \end{array}$$

(17) $$\begin{array}{cc} \sum_{j\in N_{i}}x_{ij}=y_{i}\; & \forall i\in S, \end{array}$$

(18) $$\begin{array}{cc} \sum_{i\in N_{j}}x_{ij}=y_{j}\; & \forall j\in S, \end{array}$$

(19) $$\begin{array}{cc} x_{ij},y_{i}\in \left\{0,1\right\}\; & 1\leq i,j\leq n. \end{array}$$

Notations used in above equations are listed in Table 2. Equations (5) and (6) are acoustic-channel interference constraints. Because of the interference in acoustical communication, each sensor can only transmit to or receive from another sensor through a specific acoustic channel at a time (Equation (5)). We use a binary variable $\chi_{ij}^{m}\left(t\right)$ to indicate the sub-channel state. If node *i* transmits data to node *j* on sub-channel *m* at time *t*, $\chi_{ij}^{m}\left(t\right)=1$. Otherwise, $\chi_{ij}^{m}\left(t\right)=0$. In Equation (6), $I_{m}^{j}$ is the sensor set within the *j*th sensor’s interference range on channel *m*. If sensor *j* is within the interference range of sensor *k* on sub-channel *m*, and sensor *k* is transmitting data to its neighbor *l* via *m* sub-channel, then sensor *j*’s neighbor *i* will fail to transmit to sensor *j* through sub-channel *m* at this time because of signal interference (Equation (6)).

Equation (8) to (14) are data flow constraints. We define $S_{A}$ and $S_{U}$ to represent the sensor sets that will be visited by AUV and will not be visited by AUV, respectively. Then there is no data flow to $S_{U}$ sensors (Equations (8) and (9)) and no data flow from $S_{A}$ sensors (Equations (10) and (11)). Moreover, the data flow from an $S_{U}$ sensor equals the data it sensed (Equation (12)), and the outgoing data of each sensor is the sum of the incoming data and its sensing data (Equation (13)). Variable $v_{i}$ in Equation (12) is the sensing rate of sensor *i*. In addition, the data flow on each sub-channel should not exceed the link capacity (Equation (14)). Variable $c_{ij}^{m}$ in Equation (14) is the channel capacity between sensor *i* and sensor *j* on sub channel *m*.

Equation (15) means that, during the data gathering process, each node should not spend more energy than its remained energy. Variable $d_{i}$ in Equation (16) is the buffer size of the *i*th sensor, and Equation (16) constrain the data amount stored on an $S_{A}$ sensor should not exceed the buffer size of this sensor. A binary variable $y_{i}$ is used to indicate the sensor type. If sensor *i* is an $S_{A}$ sensor, then $y_{i}=1$. Otherwise, $y_{i}=0$. Equations (18) and (19) are the travel tour constraints that each chosen sensor must visit exactly once. In addition, the AUV stop time on each sensor for data gathering is:

(20) $$t_{i}=\frac{v_{i}\Delta t-\sum_{j\in N_{i}}D_{ij}+\sum_{j\in N_{i}}D_{ji}}{m},\forall i\in S.$$

Since there is a polynomial-time reduction from the traditional traveling salesman problem to the above problem by setting $p_{i}^{in}$ and $p_{i}^{out}$ to zero, the above problem is an non-deterministic polynomial-time hard (NP-hard) problem. We implement the problem using Matlab+YALMP+GUROBI and found that, for a small scenario with no more than eight nodes, it takes more than an hour to find the optimal solution. Considering that this problem is NP-hard, the execution time would grow exponentially, and we propose a heuristic algorithm $HAS^{4}$ to solve this problem in the following section.

## 4. Centralized and Distributed Algorithms

In this section, we provide HAS${}^{4}$ to solve the MILP, and give a centralized and a distributed version of HAS${}^{4}$.

### 4.1. Description of HAS${}^{4}$

Since the path planning problem is well studied as the traditional travelling salesman problem (TSP) in previous works [28], in this paper, we focus on a sink sensor selection problem. In HAS${}^{4}$, we define $S_{C}$ to represent the sensor set that needs to be confirmed as to whether it will be visited by AUV. At first, all sensors are in $S_{C}$ and then HAS${}^{4}$ determines which sensor falls into $S_{A}$ and which sensor falls into $S_{U}$. During this process, HAS${}^{4}$ greedily chooses sensors with less energy to $S_{A}$. This is because acoustic transmission consumes more energy than acoustic reception and MI communication. By selecting these sensors to $S_{A}$, in this round of data gathering, these sensors could spend less energy to balance the network energy consumption and prolong the network lifetime.

For the objective of minimizing the AUV cost, the mobile AUV should visit as few sensors as possible. For the purpose of minimizing the network energy consumption, the network should reduce acoustic communications, which means that AUV should visit as many sensors as it can. It is easy to observe that these two objectives are opposite to each other. In HAS${}^{4}$, we define a variable to make a trade-off between these objectives. We provide both centralized and distributed version of HAS${}^{4}$. Centralized HAS${}^{4}$ can leverage all sensors’ information about remainder energy, data sensing rate, channel capacity, etc. to make a sub-optimal solution for the problem in Section 3.2. Although the surface station or AUV could be the centralized decision maker, with the long propagation delay of acoustic communications and slow speed of AUV, centralized HAS${}^{4}$ may not scalable for large underwater networks. Distributed HAS${}^{4}$ utilizes the information from sensor-self and its one-hop neighbours to decide which set this sensor belongs to. Thus, it is very practical with both small and large size networks.

### 4.2. Centralized HAS${}^{4}$

In centralized HAS${}^{4}$, the trade-off factor *l* falls in [0,1], and 1 – *l* is the proportion of $S_{U}$ candidate in all sensors, which means at least n×l sensors will be visited by the mobile AUV. The whole procedure is as shown in Algorithm 1.

Firstly, HAS${}^{4}$ estimates the lifetime of each sensor to heuristically sort the sensors. The sensor lifetime is defined as the value that the remainder of the energy divides by the energy consumption in this period. The energy is consumed on data sensing and transmission, and sensing consumption is almost constant with the preassigned task. Since the energy spent on transmission varies between different modes and acoustic is much costlier than MI, without loss of generality, we use acoustic transmission cost as the communication energy consumption. In addition, due to the fact that we have not known the data flow, we average all possible values as the consumption. After sorting the sensors, a lifetime threshold is set as the lifetime of the ⌈nl⌉-th sensor that sensors with smaller lifetimes can not be chosen into $S_{U}$.

Secondly, HAS${}^{4}$ greedy selects the minimum lifetime sensor from $S_{C}$ into $S_{A}$, and then adds all its neighbours into *U*. We denote *U* as the tuple set of candidate sensors of $S_{U}$ with its upper level sensor. The upper level sensor is the sensor where the sensing data would transmit to.

Thirdly, HAS${}^{4}$ heuristically chooses the sensor with the largest lifetime from *U* to verify if it could be added to $S_{U}$. In the verification, HAS${}^{4}$ compares its lifetime with the threshold and all constraints in Section 3.2. If it satisfies all conditions, HAS${}^{4}$ adds this sensor to $S_{U}$, changes channel indicators and adds its neighbours from $S_{C}$ to *U*. Otherwise, it removes this sensor from *U*. HAS${}^{4}$ repeats this process until *U* is empty and then goes to the second step.

When $S_{C}$ is empty, the shortest tour among all $S_{A}$ could be solved by approximately solving a TSP.

Since the outer while loop will execute at most n times, where *n* is the node number, and the inner while loop will execute at most |U| times, the centralized HAS${}^{4}$ is a Ω(mn) time algorithm, where *m* is neighbour number of the node with maximal neighbours.

**Algorithm 1:** Centralized HAS${}^{4}$.

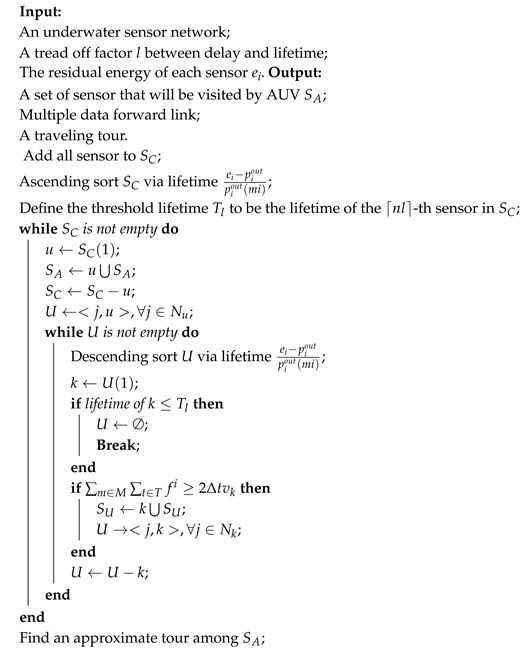



### 4.3. Distributed HAS${}^{4}$

The distributed HAS${}^{4}$ is a multi-round procedure. Each round consists of the following stages: self-elect inquiry, self-elect affirm, root select, root affirm, channel-use broadcast and conflict handler. Sensors serving different roles participate in different stages and show different behaviors in each stage. Moreover, in distributed HAS${}^{4}$, the trade-off factor *l* indicates the maximum acoustic path from any $S_{U}$ sensors to $S_{A}$ sensors.

In distributed HAS${}^{4}$, each $S_{C}$ node changes itself to be $S_{A}$ node with probability 1/lifetime. When there are some $S_{A}$ nodes, the relationship between $S_{A}$ and $S_{C}$ becomes a matching problem. Each $S_{C}$ node takes the distance to each $S_{A}$ node and the lifetime of each $S_{A}$ node as the preference to choose a root node. Each $S_{A}$ accepts some $S_{C}$ nodes on the purpose of maximizing its group. Once an $S_{C}$ node joins a group, it becomes $S_{U}$ node. When there are no $S_{C}$ nodes, this round of matching problem is solved.

The execution of distributed HAS${}^{4}$ on $S_{C}$ nodes is as shown in Algorithm 2. For each $S_{C}$ node, in the self-elect inquiry stage, it temporarily changes itself to be an $S_{A}$ node with probability 1/lifetime and broadcasts a self-elect-inquiry message to its neighbours. If it does not receive any self-elect-inquiry messages from its neighbours or its lifetime is less than all its temporarily $S_{A}$ nodes, it will successfully be an $S_{A}$ node and broadcast a self-elect-affirm message. Otherwise, it will change back to $S_{C}$. Once an $S_{C}$ node receives a self-elect-affirm message, it will add this node to its root candidate set *U*. In the root select stage, an $S_{C}$ node chooses the maximum lifetime node from *U* as root, and broadcasts a root-inquire message. If an $S_{C}$ node receives a positive message in the root affirm stage, it will temporarily become an $S_{U}$ node and broadcast a channel-occupation message based on the positive message. Once an $S_{C}$ node receives a channel-occupation message, it will update its available channels. If a temporary $S_{U}$ node does not receive any channel-occupation message or it has a longer lifetime or is closer to the root, it will successfully be an $S_{U}$ node. Otherwise, it will change back to $S_{C}$ and broadcast a channel-collision message.

**Algorithm 2:** Distributed HAS${}^{4}$
$S_{C}$.

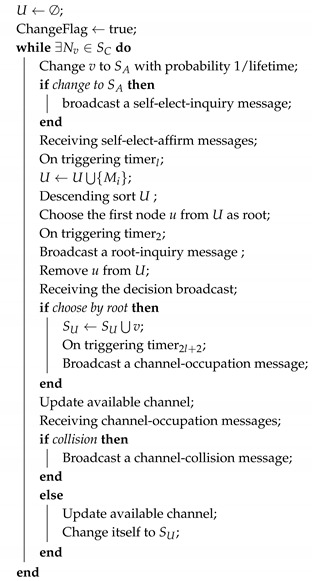



**Algorithm 3:** Distributed HAS${}^{4}$
$S_{A}$.

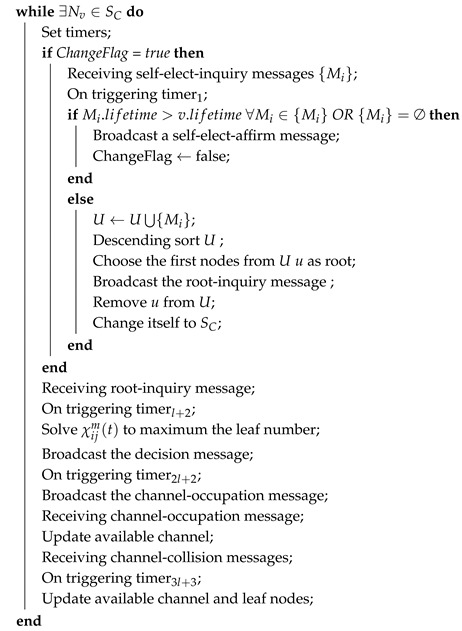



**Algorithm 4:** Distributed HAS${}^{4}$
$S_{U}$.

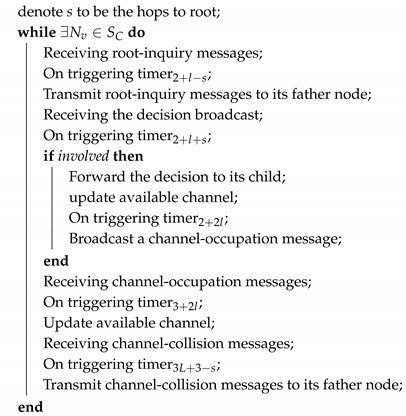



The execution of distributed HAS${}^{4}$ on $S_{A}$ nodes is as shown in Algorithm 3. For each $S_{A}$ node, it receives root-inquire messages, takes some appropriate $S_{C}$ nodes into its group according to all the constraints in Section 3.2, and broadcasts this decision. Once an $S_{A}$ node receives a channel-occupation message or channel-collision message, it will update the available channels. The execution of distributed HAS${}^{4}$ on $S_{U}$ nodes is as shown in Algorithm 4. In distributed HAS${}^{4}$, $S_{U}$ nodes are in charge of forwarding the messages between $S_{A}$ and $S_{C}$.

Since in root select, root affirm and conflict handler stages, messages from $S_{C}$ ($S_{A}$) should be forwarded hop-by-hop to $S_{A}$ ($S_{C}$) sensors, while, in the other stages, sensors just independently broadcast messages. Thus, the duration of root select stage is equal to the root affirm stage and conflict handler stage, and relates to the maximum hop in the network, which is *l* time slots. In addition, the duration of self-elect inquiry, self-elect affirm and channel-use broadcast is much shorter and equals one time slot. Thus, there are 3l+3 time slots in each round of distributed HAS${}^{4}$.

Since the message that transmitted in a distributed algorithm is not determined, the message transmitting time of each time slot should be randomly chosen in distributed HAS${}^{4}$ to reduce the collision probability. The execution of distributed HAS${}^{4}$ will exit on a sensor, when itself and all its neighbors are not within $S_{C}$.

For the worst execution of the distributed HAS${}^{4}$ that there is only one $S_{A}$ node and all node are sequentially joined in the group, the $S_{A}$ node should answer n-1 messages, and the message number for each of the rest nodes would not exceed 2n. Thus, the distributed HAS${}^{4}$ is a $\Omega (n^{2})$ message algorithm.

## 5. Simulation

### 5.1. Simulation Setting

In this simulation, sensors are randomly deployed in a 10 km × 10 km × 1 km area, and the results are from 1000 runs. The initial energy of each sensor is 40 kW·h. The transmission power of acoustical communication is 190 dB re 1 μPa (approximate 6.67 W). Since MI communication can be powered by AUV, we don’t take MI communication energy into account. The frequency of data gathering is 10 h. In addition, the data sensing rate follows the normal distribution of N(10,2) kbit/s. The benchmark in this simulation is statically using the result of first round HAS${}^{4}$’s execution.

### 5.2. Results and Analysis

**Figure 5:**
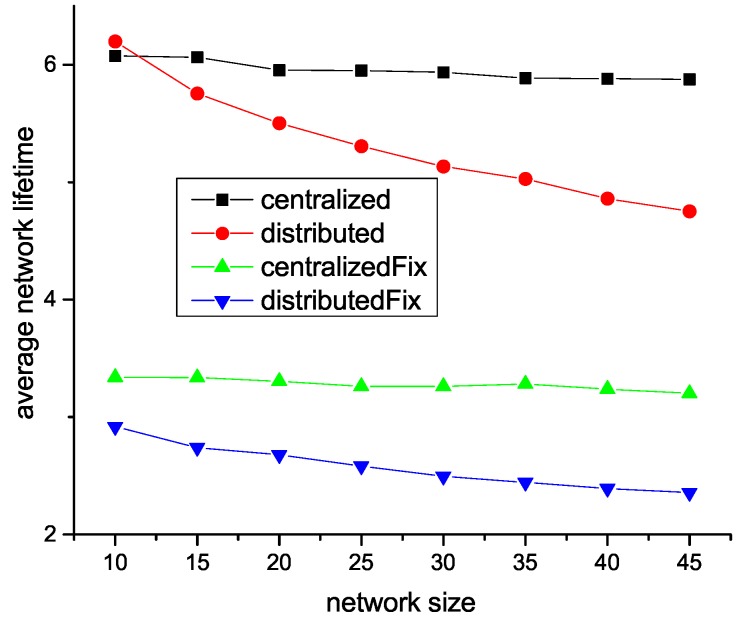
Network lifetime vs. network size with different sink selection algorithms.

As shown in Figure 5, network lifetime decreases with the increment of network size. The reason is that, in a large network, the data amount forwarded by relay nodes is much larger than a small network that consumes more energy on data forwarding. We can also observe that centralized algorithms achieve better performance than distributed algorithms. This is because centralized algorithms can leverage all sensors’ information to make a sub-optimal solution.

**Figure 6:**
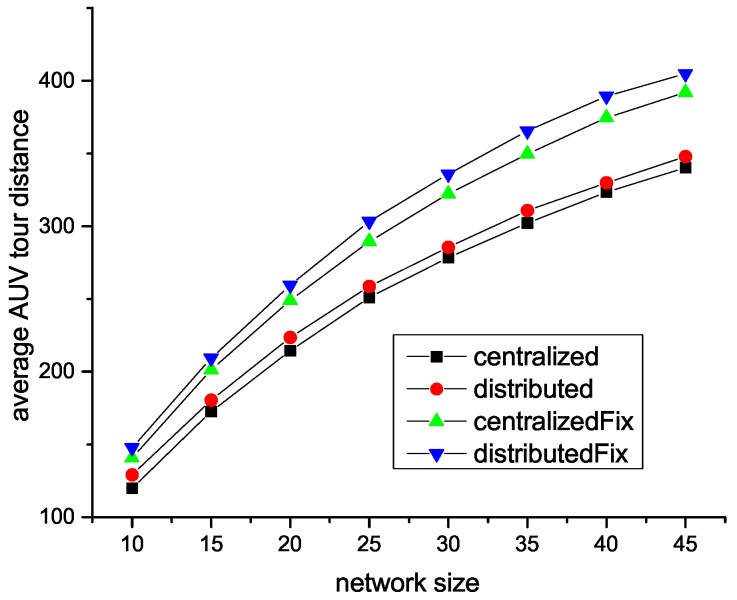
Tour vs. network size with different sink sensor selection algorithms.

Due to the channel capacity, more $S_{A}$ sensors are needed in a larger network. Thus, as shown in Figure 6, AUV travels longer in a larger network than a small one.

**Figure 7:**
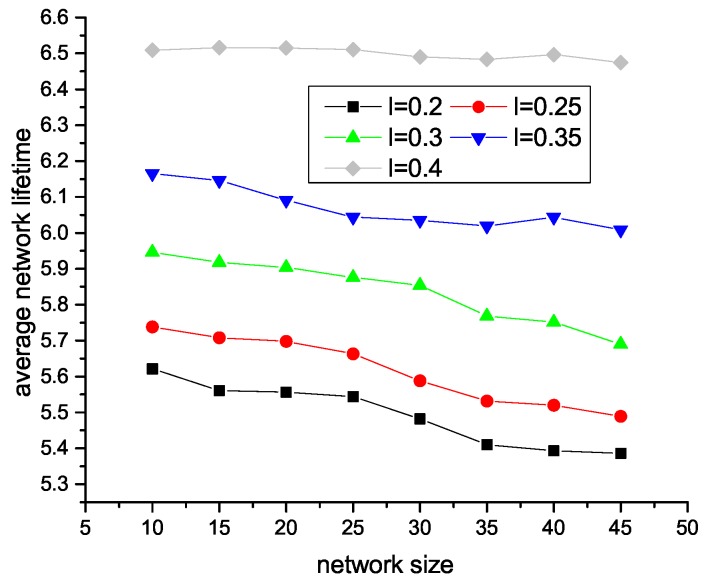
Effect of parameter *l* in centralized HAS${}^{4}$ on network lifetime.

**Figure 8:**
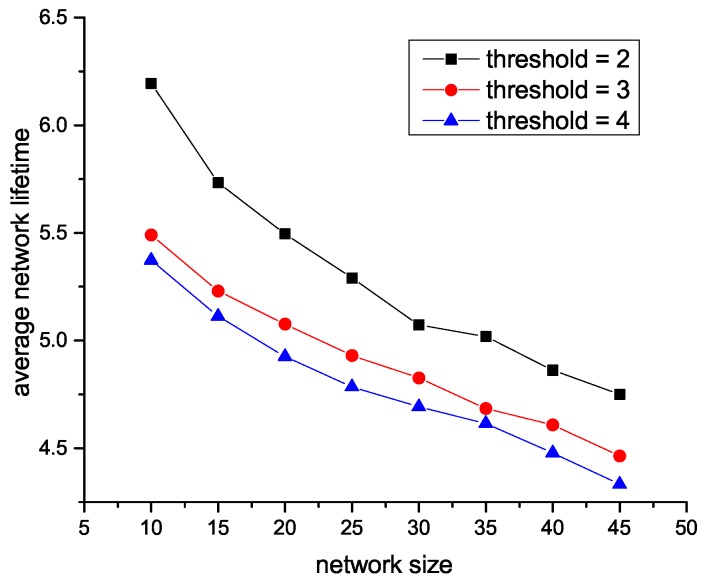
Effect of parameter *l* in distributed HAS${}^{4}$ on network lifetime.

Figure 7 and Figure 8 illustrate how the network lifetime changes with different trade-off factors in different network sizes. As we can see, in centralized HAS${}^{4}$, the network lifetime grows with the growth of trade-off factor. This is because the bigger the trade-off factor is, the less $S_{U}$ nodes exist, which reduces the amount of acoustic data communication. In distributed HAS${}^{4}$, since the trade-off factor is the longest source to end path. Thus, with a small trade-off factor, there are more $S_{A}$ nodes, which reduce the acoustic communications.

**Figure 9:**
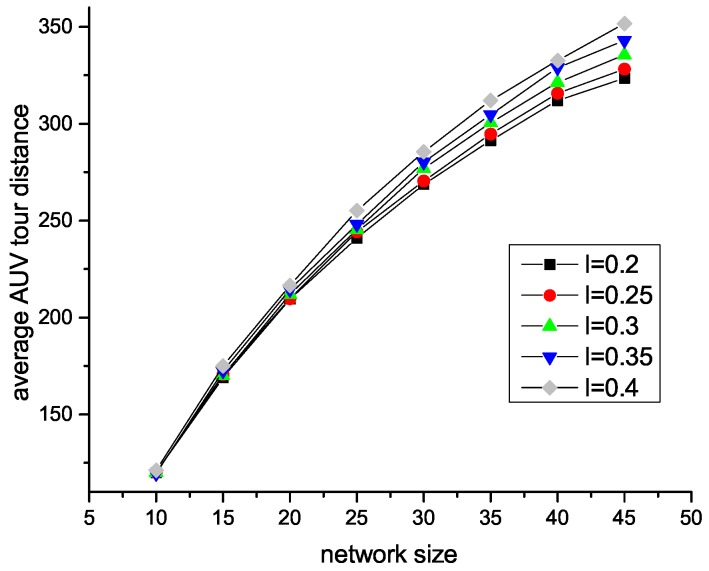
Effect of parameter *l* in centralized HAS${}^{4}$ on tour.

**Figure 10:**
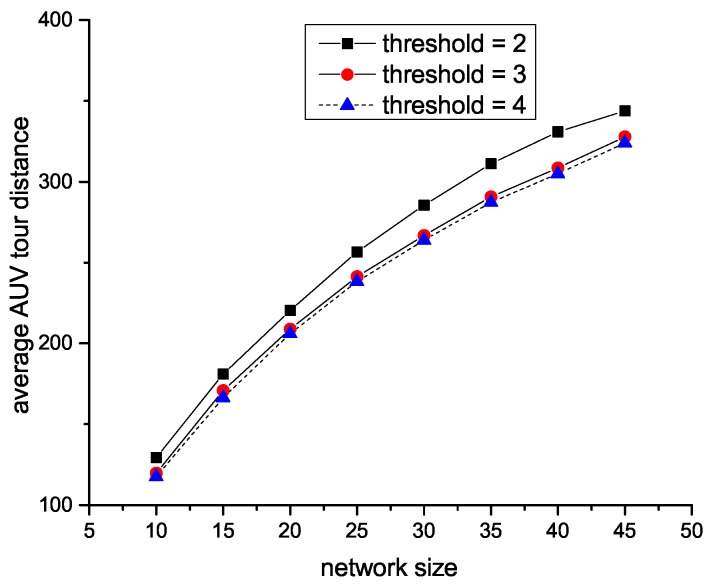
Effect of parameter *l* in distributed HAS${}^{4}$ on tour.

As we designed in centralized HAS${}^{4}$, the trade-off factor is the maximum percentage of $S_{A}$ nodes, the larger the factor is, the more $S_{A}$ nodes will be. Thus, the reason that the AUV tour increases with the increment of trade-off factor in Figure 9 is with the trade-off factor grows, $S_{A}$ nodes correspondingly increase, which leads to the increment of the average AUV tour. However, the threshold of distributed HAS${}^{4}$ is designed as the maximum acoustic path from any $S_{U}$ sensors to $S_{A}$ sensors, which results in that as shown in Figure 10, and the number of $S_{A}$ nodes will decrease with the increase of threshold.

## 6. Conclusions

In this paper, we solve the underwater data gathering problem in an efficient scenario, in which an AUV is utilized to visit part of the sensors and collect data via high speed MI communication, while the remaining nodes acoustically route their data to the ones that will be visited by the AUV. We propose both distributed and centralized algorithms HAS${}^{4}$ to select sink sensor sets in this AUV-aid data gathering applications. By setting the trade-off factor, HAS${}^{4}$ can achieve both energy and AUV efficiency.

For data gathering scenarios, the cooperation among multiple AUVs or the partition of the gathering task with high delay, unreliability, and low bandwidth acoustic communication in large scale UWSNs is much more challenging. Thus, in the future, we will further examine underwater data collection, including multi-AUV, task partition and online path planning. 

## Figures and Tables

**Table 1:** Comparison of underwater transmission modes.

Communication Method	Data Rates	Communication Ranges	Channel Dependency
acoustic	Kbps	km	Multi-path, Doppler, temperature,
			pressure, salinity, environment sound noise
electromagnetic	Mbps	10–100 m	Conductivity
optical	Mbps	10–100 m	Light scattering, Line-of-sight
			communication, Ambient light noise

**Table 2:** Notations.

$d_{ij}$	The distance between node *i* to node *j*.
$c_{i}$	The coordinate of node *i*.
$\chi_{ki}^{m}\left(t\right)$	A binary variable, that equals to 1 if node *i* transmits data to node *j* on sub-channel *m* at time *t*.
$N_{i}$	The neighbor node set of node *i*.
$D_{ik}$	The data amount from node *i* to node *k*.
$c_{ij}^{m}$	The channel capacity between node *i* and node *j* on sub channel *m*.
$y_{i}$	An indicator that equals 1 if node *i* is an $S_{A}$ node.
$S_{A}$	The node set that will be visit by an AUV.
$S_{U}$	The node set that will not be visit by an AUV.
$S_{C}$	The node set that need to be confirm whether will be visit by AUV.
